# A Keystone Ant Species Provides Robust Biological Control of the Coffee Berry Borer Under Varying Pest Densities

**DOI:** 10.1371/journal.pone.0142850

**Published:** 2015-11-12

**Authors:** Jonathan R. Morris, John Vandermeer, Ivette Perfecto

**Affiliations:** 1 School of Natural Resources and Environment, University of Michigan, Ann Arbor, Michigan, United States of America; 2 Department of Ecology and Evolutionary Biology, University of Michigan, Ann Arbor, Michigan, United States of America; Natural Resources Canada, CANADA

## Abstract

Species’ functional traits are an important part of the ecological complexity that determines the provisioning of ecosystem services. In biological pest control, predator response to pest density variation is a dynamic trait that impacts the provision of this service in agroecosystems. When pest populations fluctuate, farmers relying on biocontrol services need to know how natural enemies respond to these changes. Here we test the effect of variation in coffee berry borer (CBB) density on the biocontrol efficiency of a keystone ant species (*Azteca sericeasur*) in a coffee agroecosystem. We performed exclosure experiments to measure the infestation rate of CBB released on coffee branches in the presence and absence of ants at four different CBB density levels. We measured infestation rate as the number of CBB bored into fruits after 24 hours, quantified biocontrol efficiency (BCE) as the proportion of infesting CBB removed by ants, and estimated functional response from ant attack rates, measured as the difference in CBB infestation between branches. Infestation rates of CBB on branches with ants were significantly lower (71%-82%) than on those without ants across all density levels. Additionally, biocontrol efficiency was generally high and did not significantly vary across pest density treatments. Furthermore, ant attack rates increased linearly with increasing CBB density, suggesting a Type I functional response. These results demonstrate that ants can provide robust biological control of CBB, despite variation in pest density, and that the response of predators to pest density variation is an important factor in the provision of biocontrol services. Considering how natural enemies respond to changes in pest densities will allow for more accurate biocontrol predictions and better-informed management of this ecosystem service in agroecosystems.

## Introduction

The functional traits of species play a major role in the provisioning of ecosystem services [1]. While much of the ecosystem service literature has focused on the influence of species richness on ecosystem service provision, it is ultimately species’ traits that drive ecological processes [2–4]. However, the way a species functions is often context dependent on environmental conditions, so the effectiveness of an ecosystem service provider may change if ecological conditions change. This complexity has been noted as a research priority for the ecological study of ecosystem services [5,6], yet not enough has been done to test this idea. Attention to species’ traits and the dynamics of ecosystem service provision is especially important in agricultural systems [7,8], where farmers relying on these services need to know how to manage for them over the course of growing seasons.

Biological pest control is one of the most widely recognized ecosystem services provided by biodiversity in agriculture [7,9]. Where natural enemies can effectively limit crop pests, farmers can reduce or potentially eliminate their reliance on chemical pesticides while maintaining high levels of production [9–11]. Successful realization of this ideal in agroecosystems, however, depends on a complex array of ecological conditions [12,13]. The traits of natural enemies, ranging from hunting mode to prey selectivity [14,15], can play a large role in determining how biocontrol will function [16,17]. Of particular importance is how predators respond to changes in prey density [16], which occurs through two principle mechanisms. The first is predator functional response, which is a measure of how a predator’s attack rate changes in response to increasing prey density. This has been an important component of classical predator-prey theory ever since Holling’s original work [18–20], where he describes three general functional response curves: Type I, a linear increase in attack rate; Type II, an increase in attack rate that gradually plateaus; Type III, an initial lag, followed by an acceleration, then leveling off of attack rate, resulting in a sigmoidal curve. Most commonly, predators exhibit either Type II or Type III functional response curves, where attack rates gradually level off with increasing prey density as they either satiate or are overwhelmed in their handling time of individual prey [20–22]. Where this leveling off occurs can determine the stability of predator-prey interactions, whether or not pests escape control, and ultimately how effective a biocontrol agent will be in suppressing crop damage [20,21]. The second mechanism through which predators respond to prey density is numerical response, or how predator density changes with changing prey density. This can occur over short time-scales where individual predators move to areas of increased prey (aggregation numerical response), but can also occur over longer time-scales, where predators reproduce more as they consume increasing prey densities and, in turn, increase their own densities (reproductive numerical response) [16,23]. Overall functional and numerical response can work together or independently to determine whether predators will be successful in suppressing pest outbreaks and reducing crop damage, thus making the response of predators to prey density an important factor in the provisioning of biological control services [16,20,22,23].

Coffee agroecosystems are well-suited venues for exploring how components of ecological complexity, like context dependent predator traits, impact ecosystem service provision [13,24]. Coffee is one of the most important global commodities, and with nearly 20 million farming households around the world, its production provides widespread economic benefits to society [25]. Throughout the tropics, coffee plantations threaten biodiversity with habitat loss, as they are often located in some of the world’s most important biodiversity hotspots [26], but they can also help to conserve it by providing high quality habitat patches within an intensive agricultural matrix [27]. The latter is especially true in traditional shade-grown coffee systems where shade trees offer nesting space and resources to native biodiversity [28]. Because of this, much attention has been given in the literature to the ecosystem service potential of biodiversity in these agroecosystems, with a particular focus on biological pest control [24,29].

Much of the biocontrol work in coffee has focused on the coffee berry borer (*Hypothenemus hampei*, Ferrari, Coleoptera: Curculionidae) a notorious insect that is considered the most economically damaging pest of coffee throughout the world [30,31]. Adult females of this small beetle bore directly into the coffee fruit, where they carry out their reproductive cycle, laying eggs, which develop into larvae that eat the seed. Borer infestation reduces the quality of the coffee crop, often ruining the berries, which can result in yield losses of over 30% in some regions [32]. Furthermore, because of its cryptic life cycle inside the berries, it is difficult to control with pesticides, and when chemical control is practiced it is often done with toxic insecticides, such as endosulfan [30,33]. Fortunately, a number of natural enemies of the coffee berry borer (CBB) have been identified, many of which are native to their respective coffee growing regions [31,34–46]. These species offer the potential for conservation or autonomous biological control [12,47], where farmers can manage these services indirectly by bolstering natural enemy populations through the maintenance of complex habitat in and around coffee farms [12,29,37]–potentially resulting in win-win solutions for both farmers and biodiversity [10,17,48].

Many of the CBB natural enemies that have been studied are ants [38–46], which have long been used as biological control in agriculture [49–51]. Ants can reduce CBB infestation and damage on coffee plants both directly through predation and indirectly through plant defense–where they engage in protective relationships with plants, removing herbivores in exchange for resources such as nesting space or honeydew from tending hemipteran insects [45,46]. Despite this knowledge across a diversity of ant species, we still know little about how well ants serve as pest control agents under changing pest density conditions. Few studies have explicitly tested this question with ants, perhaps because of their uniqueness as generalist eusocial predators. The colonial nature of ants may make it logistically difficult to directly measure their functional response. One study attempted this with individual *Solenopsis invica* workers (separated from their colony) exposed to cotton fleahopper pests in the laboratory [52]. However, as Schenk and Bacher (2002) suggest, field tests of functional response are preferred for generalist predators as they incorporate the impact of alternative prey items and the potential for prey-switching–where generalist predator attack rates accelerate as they switch to more abundant prey, resulting in a Type III curve [22,53]. Additionally, testing individual workers ignores the essential nature of ants–their colonial makeup–which may ultimately explain their efficiency as biocontrol agents [54]. If ants are to be relied on as effective alternatives to chemical pesticides, farmers need to know how they respond to expansions in CBB densities under field conditions. This knowledge will help inform the management of these biocontrol agents in complex coffee agroecosystems.

**Fig 1:**
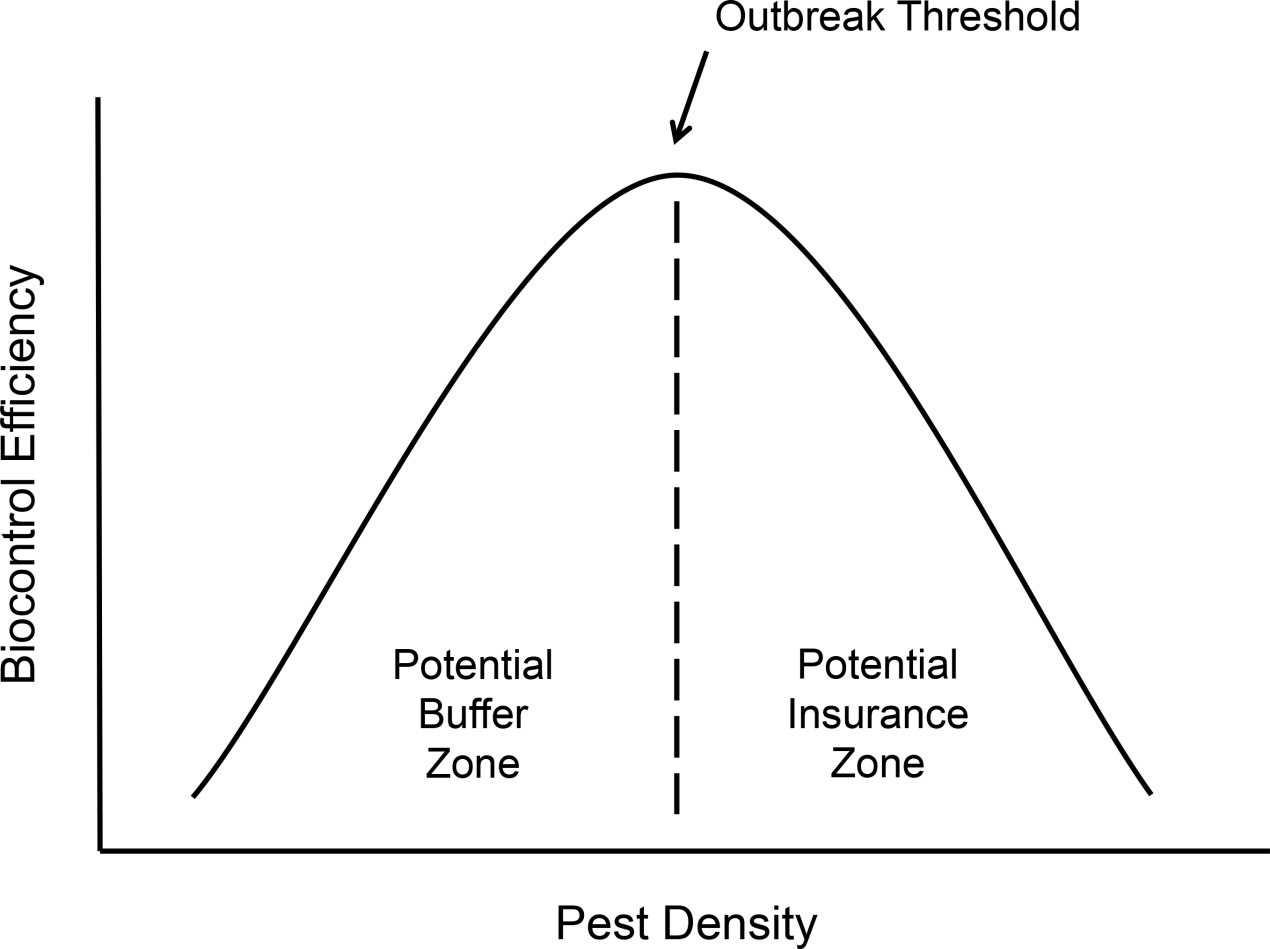
Hypothesized biological control efficiency (BCE) dynamics. As pest density increases, the overall efficiency of natural enemies to control pest infestation will likely vary, resulting in zones with different management implications. Here we define biocontrol efficiency (BCE) as the proportion of pests that are prevented from infesting or damaging crops by a predator. In our study we expect BCE to initially increase with increasing pest density, perhaps as ants switch to the more abundant prey. This could result in a potential buffer zone where ants help to buffer the pest outbreak. Eventually, however, BCE may drop off, as ants satiate or become overwhelmed at higher pest densities. This could create a potential pest outbreak threshold, which would result in an insurance zone where farmers would need to rely on the insurance of other natural enemy species to compensate for the decreased biocontrol efficiency.

Here we test how a keystone ant species, a known biocontrol agent of the coffee berry borer, responds to variation in CBB density on coffee plants. Because of the difficulties of teasing apart functional and numerical responses in ants we test this question in two ways. Through an ant exclosure experiment, we measure ant biocontrol efficiency (BCE) as the proportion of infesting pests removed from plants by ants and use this to gage the ultimate effect of ants on reducing crop damage of CBB. We also estimate ant functional response, where we consider the short-term, collective response of groups of ants on coffee (representing the colony as an individual predator). We predict that ants, as generalist predators, are likely to exhibit prey-switching but will eventually satiate, resulting in a Type III functional response curve [22,53]. Functional response and BCE are then intimately linked, where the type of functional response curve will influence how efficient ants are at different prey densities. Therefore, we predict that under changing pest densities BCE will be dynamic, where a Type III functional response would likely generate a bell-shaped BCE curve, and that this will have important implications for farmers managing for ant biocontrol services (Fig 1). Finally, we use ant activity, measured during the experiment, as a proxy for ant abundance to conduct a post-hoc estimation of numerical response. We use these measures, together, to gain some clarity on the question of how ants respond to dynamic pest densities and to determine if ant biological control can be robust under pest density increases or if this service declines. To our knowledge, this is the first reported field study to test the effect of pest density variation on ant-mediated biological control.

## Materials and Methods

### Study System & Site Selection

This research was conducted from June 25—August 14, 2014 at Finca Irlanda, a 280-hectare coffee farm in the Soconusco region of Chiapas, Mexico with permission from the farm’s owner, Don Walter Peters. Finca Irlanda is an organic, shaded coffee farm situated at 15°11' N 90°20' W, between 950 and 1150 meters, where a number of ant species occupy coffee plants and shade trees. *Azteca sericeasur* J. Longino is a dominant arboreal ant species in this system that nests on shade trees and tends scale insects on coffee bushes [40,55]. This relationship, between the coffee, scale, and ants, forms the basis of a complex web of ecological interactions, with cascading effects on a number of other coffee pests and their predators, making *A*. *sericeasur* a keystone ant species in this system [12,13]. The primary insect pest of coffee in the region is the coffee berry borer [56]. Throughout the farm, *A*. *sericeasur* is known to protect coffee from CBB both through plant defense and direct predation [45,46]. To select our sites, we surveyed the farm for 20 individual coffee bushes (*Coffea arabica*) with *A*. *sericeasur* under several conditions. First, we looked for a minimum level of ant activity on each bush to ensure ants were consistently active at the site and would likely remain over the course of the experiment. We measured bush ant activity as the number of individual ants passing a point on the central trunk per minute and set the minimum at 10 individuals/minute. We then counted the branches and berries and chose only bushes with a minimum of eight branches, each with at least 40 non-infested berries. Finally, bush replicates were chosen with at least 5m between them to increase the probability that ants on each bush represented different ant colonies.

### Branch Exclosure Experiment

**Fig 2:**
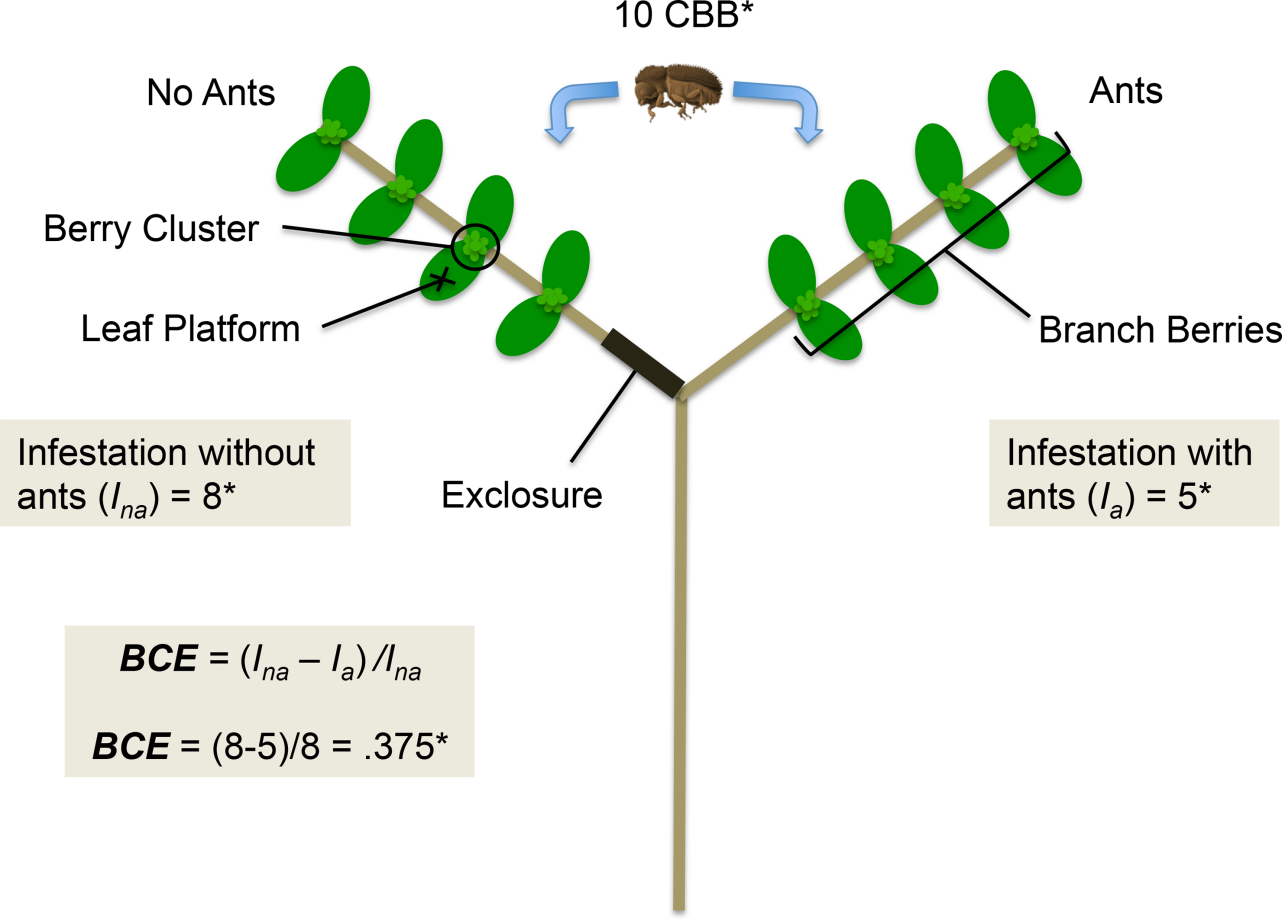
Exclosure experimental design. Shows paired-branch treatment design and calculation of biocontrol efficiency (BCE). Infestation was measured as the number of bored coffee fruits on branches 24 hrs after coffee berry borer placement. Total coffee berries per branch, leaf platforms, and berry clusters near leaf platforms are illustrated. This design was repeated on four different branch pairs within each coffee bush replicate, at different times, using four levels of CBB density. Asterisks indicate data is hypothetical and is only intended for the purpose of demonstrating the BCE calculation.

To test the effects of varying CBB density on ant biocontrol we conducted an ant exclosure experiment adapted from a similar design used by Gonthier et al. (2013) [46]. We used a paired-branch treatment design to estimate the effect of ants on CBB infestation while controlling for external variables (Fig 2). Within each coffee bush replicate, we choose pairs of branches of roughly the same age (similar height on central trunk) to control for within plant differences. We measured branch ant activity as the number of individuals on or crossing onto a branch per minute, and chose branches with at least one ant/minute. Before running the experiment, we removed all berries infested with CBB and any decaying berries from the branches that were likely to fall off during manipulation. We then removed any bridging plant material on the branches to decrease the chance of an exclosure breach. To standardize the number of berries between branch pairs we removed excess berries on branches until the difference in berries between the two branches was no greater than five (making sure to maintain the 40 berry count minimum). Next, we marked two leaves on each branch to serve as platforms for the placement of CBB individuals. We used two leaf platforms per branch to spread out the beetles to reduce any potential density effects they might have on each other. We then counted the number of berries in berry clusters nearest to the leaf platforms and removed berries, if necessary, until the difference in the sum of berries nearest to the two platforms was no greater than two between the two branches. After initial branch preparation, we randomly assigned one of the two branches as the exclosure treatment and applied tanglefoot (The Tanglefoot Co., Grand Rapids, MI, USA) around the base of the branch to prevent ants from passing onto it. We then removed all ants beyond the tanglefoot to ensure that this branch served as a non-ant treatment.

CBB were collected from infested fruits found on coffee plants in the farm. On the morning of placement, we separated CBB from fruits in the laboratory and placed them in glass vials. We were careful to only use mature females (the individuals likely to bore) based on their size and color [32]. After completing the site setup we waited a minimum of 20 hours before returning to place the CBB. We did this to allow the system to relax to the baseline level of ant activity (not artificially disturbed) and to allow any plant volatiles released during setup to dissipate. Additionally, we continued to monitor branch ant activity (number of ants on or crossing onto branch/minute) throughout the experiment to ensure that activity did not change as a result of the experimental manipulation. Upon returning to the site, we roughly split the CBB individuals between the two leaf platforms on each branch. After placing CBB on the branches, we waited 24 hours to count the level of infestation on the branches. Infestation was measured as the number of berries on each branch with CBB bored into the fruit (Fig 2). We included berries with beetles bored halfway or more into entrance holes, but not berries with empty entrance holes, as this could indicate that ant attack may have occurred during boring. While typically only one beetle enters a berry [31], in cases where berries had multiple bored holes they were only counted as one.

This process was repeated within each bush replicate on four different branch pairs using CBB densities of 10, 20, 40, and 80 individuals per branch. We chose these levels to ensure that ants were exposed to a broad range of agriculturally relevant densities, where 80 individuals per branch is meant to simulate outbreak levels based on what has been documented in Latin American coffee production [33,56]. To separate the effects of different CBB densities on ants, each density treatment was conducted at separate times within the same coffee bush replicate and the order of the treatments was chosen randomly to minimize the potential for habituating the ants to a particular pattern of pest density variation. Between conducting each density treatment within a bush we waited at least 20 hours and removed tanglefoot as often as possible from branches already used to avoid disturbing the foraging space of the ants too drastically over the course of the experiment. All treatments were initiated between 9:00 and 14:00 (before the afternoon rainy period) and ran for 24 hours.

### Biocontrol Efficiency and Functional Response

We calculated biocontrol efficiency (BCE) as the proportion of infesting CBB removed by the ants (Fig 2). This was measured as the ratio of the difference in infestation between paired branches, with and without ants, and the infestation on the branch without ants alone (where *I*_*na*_ is infestation without ants and *I*_*a*_ is infestation with ants):

$$BCE=\frac{I_{na}-I_{a}}{I_{na}}$$

This yields a ratio that typically falls between 0 and 1, with 1 meaning ants are 100% efficient (i.e. they remove all of the infesting CBB present). To estimate functional response we measured the attack rate (AR) of the ants as the difference between infesting CBB individuals between paired branches, with and without ants, or simply the numerator of the BCE equation:

$$AR=I_{na}-I_{a}$$

This yields the collective attack rate of the group of ants on a branch (representing the colony as an individual predator), and allows for an indirect estimation of functional response. Here BCE serves as a useful metric alongside functional response for assessing the relative efficacy of biological control. While functional response demonstrates the dynamics of predator attack rate under prey density variation, it does not convey the proportional amount of infesting or damaging prey that are removed by a predator. Calculating biocontrol efficiency in this way allows us to evaluate the efficiency of biocontrol agents in terms of their ultimate contribution to the suppression of crop damage.

### Statistical Analysis

To account for random effects, covariates, and non-normal data, we used a generalized linear mixed model (GLMM) [57] to test for differences in CBB infestation rates between treatments. We ran the GLMM with a log link function and a Poisson distribution, and included ant presence/absence, density treatment (four levels as a categorical variable), and their interaction as fixed effects. To control for environmental variation, ant colony differences, and non-independence between branches, we included coffee bush in the model as a random effect. We also included branch ant activity (measured before experimental set up), the number of berries per branch, and the sum of nearest berry clusters per branch as fixed effect covariates in the model. To account for the potential by-products of experimentally manipulating plants, such as the release of volatile chemicals that may have influenced CBB infestation or ant behavior, we included the total number of berries removed per branch during set up as an additional covariate in the model. To test for significant differences in BCE between the density treatments, we calculated estimated BCE means from the infestation coefficients generated by the GLMM. We then made pair-wise comparisons of these estimated BCE means based on the GLMM output.

To estimate the type of functional response from mean ant attack rates across density treatments, we fit the data to a simple linear regression model with the origin as the intercept. In order to differentiate any effects of numerical responses in the ants, we performed a post-hoc analysis of ant density (using ant activity as a proxy for density) in relation to CBB density. For this we ran a simple linear regression model of branch ant activity (on non-exclosure branches) measured at the end of the experiment (24 hours after CBB placement) to CBB density treatment level [53]. Additionally, we conducted a GLMM on mean branch ant activity (non-exclosure branches) measured over the course of the experiment. We did this to determine if ant activity varied by through time as a result of the experimental manipulation. We included activity sample time (at set up, at CBB placement, and 24 hours later at check) and CBB density treatment as categorical fixed effects, and coffee bush as a random effect.

To ensure that our density treatments were reliable tests of the effect of density variation on ants, we removed all paired-branch replicates from the analysis where infestation on non-ant branches was less than 10% of the experimental treatment density. This occurred with greater frequency toward the end of the experimental time frame, as we were unable to continue to find sufficient healthy CBB individuals in the field. Additionally, any replicates where ant activity was greater than 1 individual/minute on non-ant branches after the experiment was run were also eliminated from the analysis. This only occurred in three replicates where ants had breached exclosures, or falling plant debris had caused exclosures to fail. Together, this resulted in paired-branch sample sizes for each density treatment of: *n* = 20 for 10 CBB, *n* = 18 for 20 CBB, *n* = 15 for 40 CBB, and *n* = 14 for 80 CBB. Removing these data from the analysis did not change the overall statistical conclusions of the experiment. GLMMs were implemented using the lem4 package, tested for overall significance using Wald Type II chi-square tests, and performed–along with all other statistical tests–in R (R Development Core Team 2014) [58].

## Results

**Fig 3:**
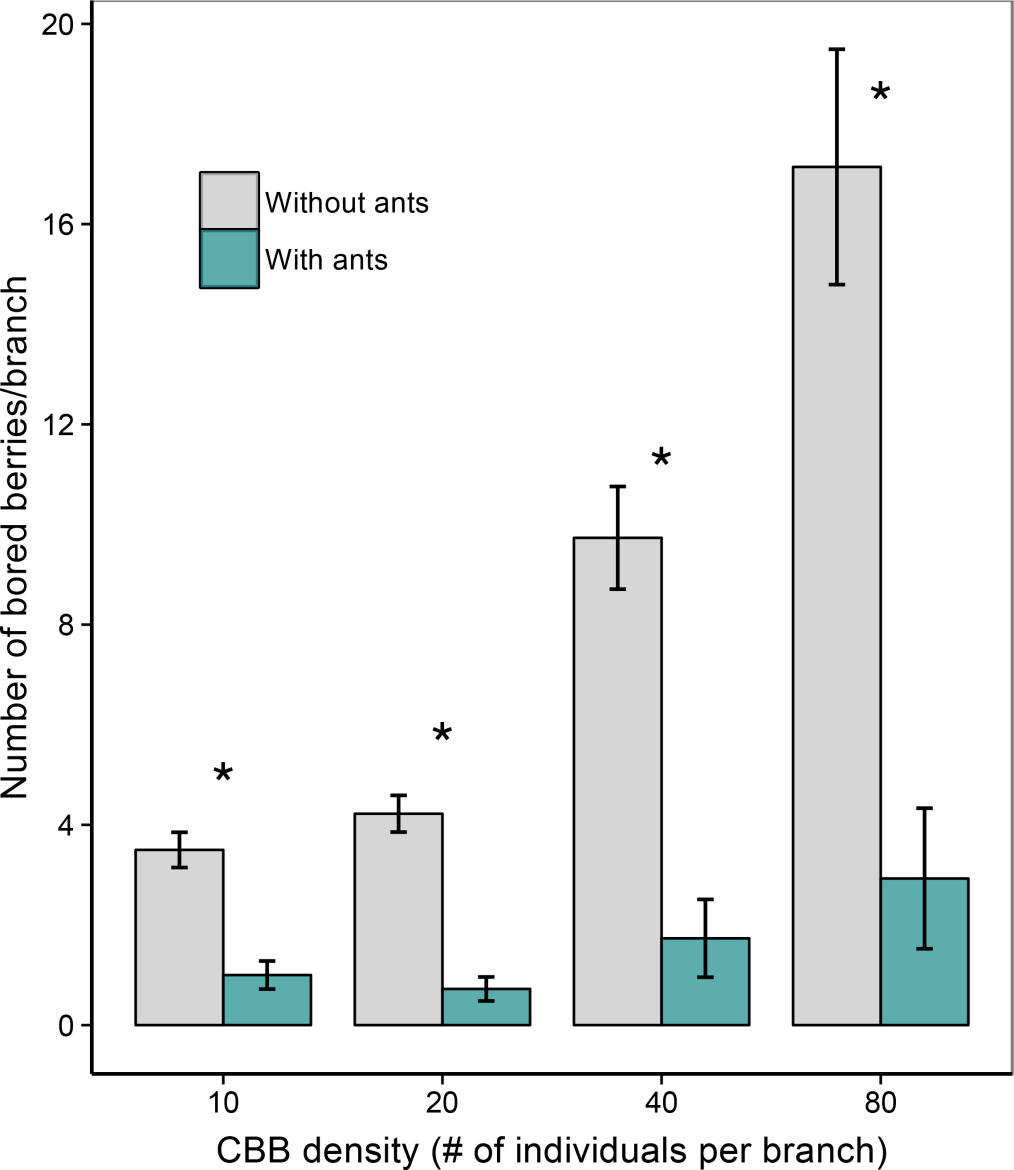
Coffee berry borer (CBB) infestation. Bars show the mean number of bored berries per branch (± SE) in the presence and absence of *Azteca sericeasur* at each CBB density treatment after 24 hours. Statistically significant differences in infestation between branches with and without ants are marked, where * = *p* < 0.001

**Fig 4:**
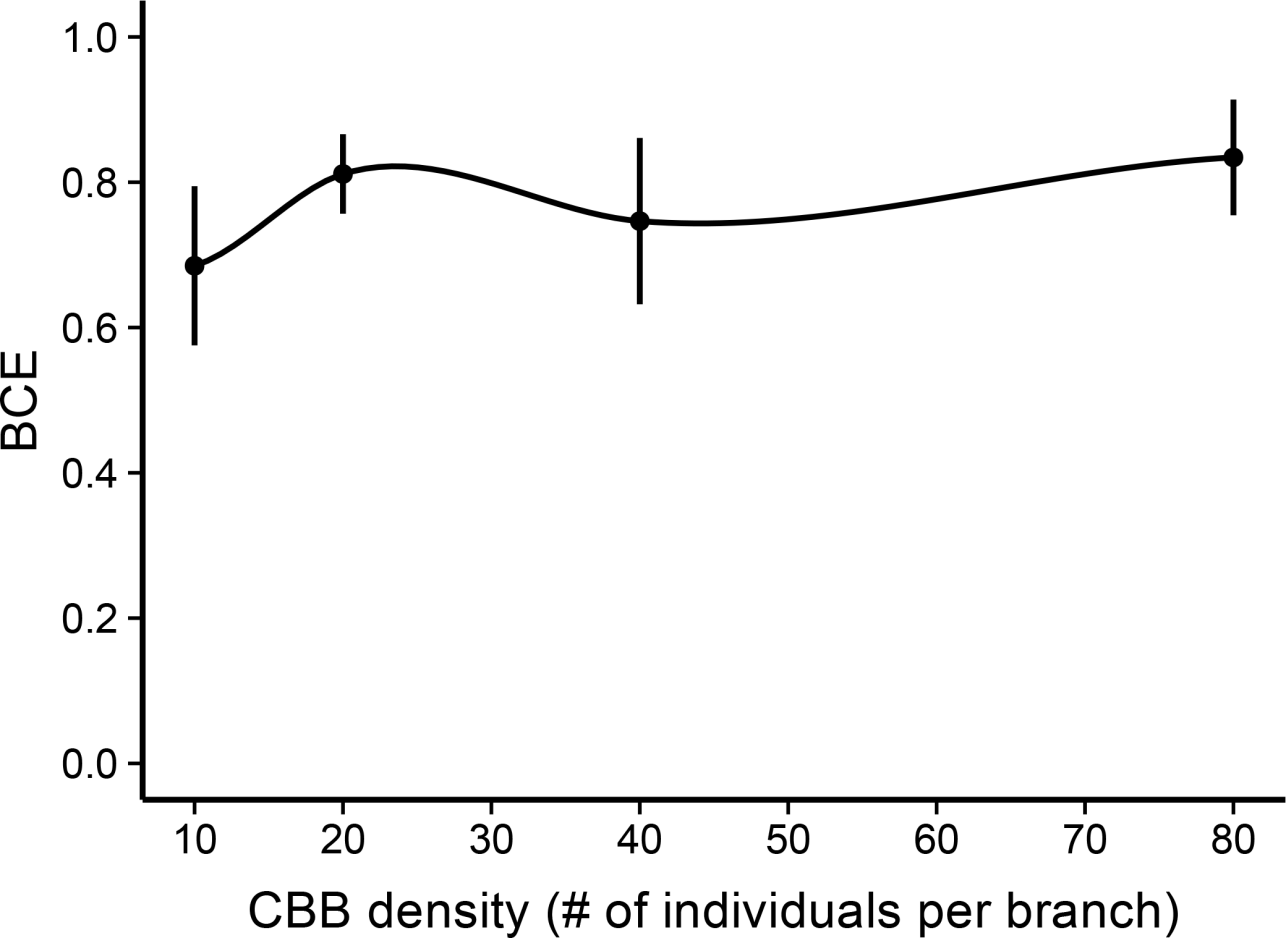
Biocontrol efficiency (BCE) curve. Shows mean BCE (± SE) of *Azteca sericeasur* at each coffee berry borer (CBB) density treatment (10, 20, 40, and 80 individuals). There was no statistical difference in mean BCE across the treatments. The curve illustrates that BCE is maintained at a high level; however, trends between 0 and 10 CBB were not tested. The curve was produced using the “loess” smoothing function in the ggplot package in R.

Overall, the GLMM showed that both ant presence/absence (*χ* ^*2*^ = 230.31, *p* < 0.001) and CBB density (*χ* ^*2*^ = 178.34, *p* < 0.001) were significant predictors of CBB infestation, but their interaction was not significant (*χ* ^*2*^ = 3.15, *p* = 0.370). Pair-wise comparison of branches with and without ants in the GLMM showed that there was a significant difference in the mean number of bored fruits after 24 hours for every CBB density treatment (10 CBB: *z* = -4.90, *p* < 0.001; 20 CBB: *z* = -5.74, *p* < 0.001; 40 CBB: *z* = -8.10, *p* < 0.001; 80 CBB: *z* = -10.54, *p* < 0.001; Fig 3). Across the density treatments, *A*. *sericeasur* reduced CBB infestation by 71%-82%. Pair-wise comparisons of estimated BCE means in the GLMM revealed that BCE did not significantly vary between the density treatments (BCE_10:20_, *z* = -1.18, *p* = 0.238; BCE_10:40_, *z* = -1.42, *p* = 0.157; BCE_10:80_, *z* = -1.71, *p* = 0.087; BCE_20:40_, *z* = -0.011, *p* = 0.991; BCE_20:80_, *z* = -0.168, *p* = 0.867; BCE_40:80_, *z* = -0.199, *p* = 0.842; Fig 4). Overall, BCE was consistently high, ranging from .685 to .834 across the treatments. Additionally, the non-significant interaction term between ant presence/absence and density treatment further supports the idea that the effect of ants was consistent at different densities. Ant activity on the branches (measured before experimental set up), the number of berries per branch, the number of nearest cluster berries per branch, and total berries removed per branch were not significant factors in the GLMM.

**Fig 5:**
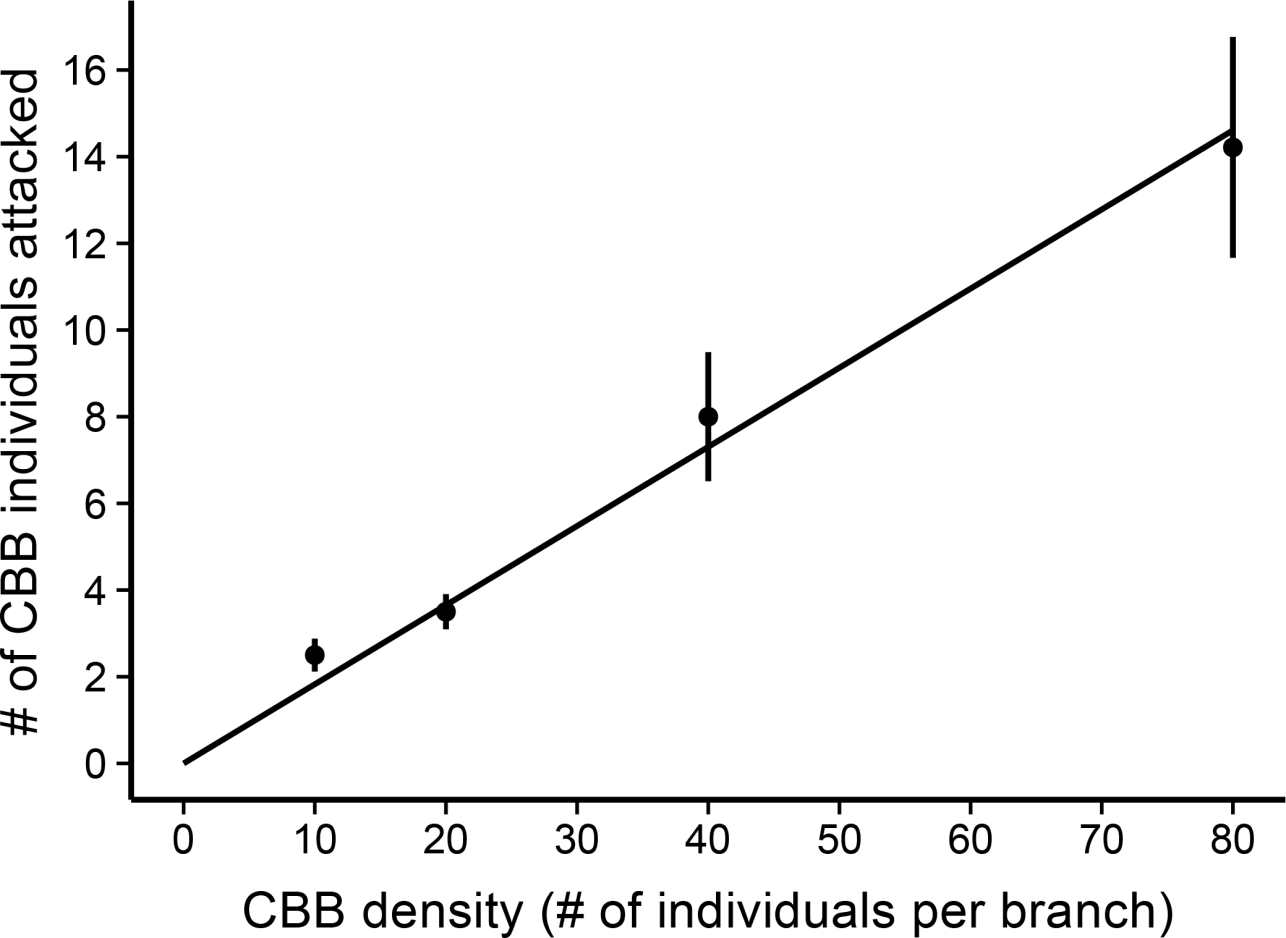
Functional response curve. Shows the mean number of coffee berry borer (CBB) individuals attacked by *Azteca sericeasur* (± SE) across CBB density treatments (10, 20, 40, and 80 individuals). A simple linear regression model, shown by the line, suggests a Type I functional response (*R* ^*2*^ = .9948, *F* = 761.4, *p* < .001).

The functional response analysis showed the data were well fit to a simple linear regression model fit through the origin (*R* ^*2*^ = .9948, *F* = 761.4, *p* < .001, Fig 5). The results indicate that the mean number of CBB individuals attacked or removed by ants increased linearly with respect to increasing CBB density, which suggests a Type I functional response in *A*. *sericeasur*. Furthermore, our post-hoc analysis of ant activity after the 24-hour experimental period showed that there was no relationship between the number of ants per branch/minute (on non-exclosure branches) and the CBB density treatment level (*R* ^*2*^ = 0.003, *F* = 0.005, *p* = 0.950, S1 Fig). This suggests there was no lasting numerical response of the ants to CBB density. Finally, our GLMM on branch ant activity (on non-exclosure branches) showed that the mean number of ants on or crossing onto treatment branches did not significantly vary during the experiment (S2 Fig). This was true in regards to sample time (*χ* ^*2*^ = 3.290, *p* = 0.193), CBB density treatment (*χ* ^*2*^ = 1.648, *p* = 0.649), and their interaction (*χ* ^*2*^ = 8.353, *p* = 0.213). This indicates that experimental manipulation did not have lasting effects on the activity level of the ants.

## Discussion

These results demonstrate that ants can be highly effective biological control agents of the coffee berry borer. Overall, the presence of *A*. *sericeasur* on coffee branches significantly reduced coffee berry borer infestation rates. This was consistent even at high levels of CBB densities and suggests that *A*. *sericeasur* could provide robust biological control in the face of future CBB outbreaks. This is supported by the consistently high level of BCE, the non-significant interaction between ant presence/absence and CBB density (suggesting consistent effect of ants), and the Type I functional response we estimated from collective ant attack rates on coffee branches. Interestingly, our failure to detect a numerical response in the ants 24 hours after CBB exposure suggests that there was no lasting effect of ants aggregating in areas of high pest density. Overall, these results conflicted with our predictions and indicate that ants, as generalist eusocial predators, may be unique in their ability to respond rapidly and robustly to increases in prey density [54]. Type I functional responses are not commonly observed in nature [16,20,22], especially within insects [21]. Typically, predator attack rates eventually level off with increasing prey density as they become overwhelmed and satiate, resulting in Type II or Type III curves [20–23]. While individual predators will eventually satiate, it does not appear that colonies of hundreds of ant workers, as *A*. *sericeasur* usually maintains, will satiate at the densities of CBB that are meaningful to farmers.

One possible explanation for this result is that *A*. *sericeasur* is not always a strict predator. Because these ants are usually protecting their scale mutualist partners on the coffee plants, they are often engaging in plant defense, where ants remove pest individuals from host plants rather than predate them [45]. While ant-plant defensive relationships most commonly benefit plants, in some cases ants can increase the density of hemipterans to pest levels [59,60]. On coffee, however, CBB directly attack the harvested crop making them a more severe coffee pest than scale insects [33]. Thus, despite the potential trade-off, ant-hemipteran mutualisms on coffee plants likely have a net positive effect [46]. This behavior may help, in part, to explain the Type I functional response we observed, as ants will not satiate unless they are consuming prey. While non-consumptive plant defense works to suppress the infestation of CBB on coffee, it may allow beetles to escape mortality. It is still likely, however, that removal of the beetle from the plant would result in the death of CBB individuals, as the insects are not very hardy outside of coffee fruits and often fall to the ground after disruption by the ants [45]. It is also possible that other species of ground foraging ants would predate beetles that are knocked off plants by *A*. *sericeasur*, as it is known that several species of ground nesting ants are predators of CBB in coffee agroecosystems [41,61]. Another possible explanation for these results is that ants may actually be storing prey individuals in their nests, which some species of ants have been known to do [62,63]. This behavior could delay the effect of satiation and would allow ants to maintain high attack rates even at very high pest densities. Whether our results were being mediated through ant-plant defense, prey storage, or are simply a consequence of ant coloniality, there is no doubt that the presence of *A*. *sericeasur* on coffee plants helps to reduce CBB infestation rates [40,45,46]. Further work on the exact mechanism of *A*. *sericeasur’s* consistently high BCE will help to better inform management of ant-mediated CBB biocontrol and will also allow for inference about the biocontrol potential of other species of ants.

More broadly, these results demonstrate the importance of considering the functional traits of ecosystem service providers and the dynamics of ecosystem service provision in agriculture [7,8]. In the case of the coffee berry borer, much uncertainty remains regarding the severity of this pest in the future. While its infestation levels are currently low in southern Mexico where this experiment was conducted, in other locations it has recently reached outbreak levels, causing serious crop losses in island producing regions such as Hawaii and Jamaica [64–66]. If CBB outbreaks occur in other locations, farmers will need to know if biocontrol services will still be effective. Indeed, recent literature suggests outbreaks may become more probable as climate change advances around the planet. Jaramillo et al. (2011) predict that increasing average temperatures in coffee growing regions will not only expand the altitudinal range of CBB, allowing it to reach higher grown coffee, but will also expand the temporal window for CBB reproductive cycles on farms, ultimately leading to increases in pest densities [67]. Furthermore, additional unexpected changes may come with CBB expansion in the form of increased fungal infestation rates associated with CBB crop damage, such as molds that produce toxic compounds, like ochratoxin A, which can contaminate coffee harvests [68]. We believe this presents an example of the importance of the insurance hypothesis of biodiversity [69]: while ants may go unnoticed at low densities of CBB, they could become very important ecosystem service providers in the face of future outbreaks. In this case, maintaining ants that are robust to pest density increases on farms would be crucial for coffee farmers relying on biological control.

As far as we know, this is the first reported field experiment to test the effect of increasing pest density on an ant species in the context of biological control. This is surprising considering the history of their use in agriculture and their widespread potential as biocontrol agents [49–51,70]. While their colonial nature may make them atypical organisms to consider in terms of functional response, they are important predators nonetheless. Indeed, the functional response that ants exhibit as a colony may help to explain their efficacy as biocontrol agents [54]. Alternatively, the use of biocontrol control efficiency (BCE) as an additional measure to functional and numerical response may allow for more direct inference about the ultimate impact of predators such as ants on reducing crop damage. Further work will need to be done to know if the trends we found generalize to other ant species or are a unique result of a keystone species engaging in aggressive plant defense. More broadly, the effect of prey density variation on natural enemies is an important part of the ecological complexity that governs the provision of biological control services, and should continue to be researched. Through experimentally studying this dynamic attribute of biocontrol ecologists will be able to better support management-relevant predictions in agriculture with empirical knowledge. This is precisely the kind of information farmers need if they are to embrace the conservation of biodiversity and ecosystem services in order to sustainably manage the world’s food supply.

## Supporting Information

S1 Dataset Dataset analyzed for this study. Includes data and meta-data.
(XLSX)

S1 Fig Numerical Response. (PDF)

S2 Fig Branch Ant Activity. (PDF)
