# Supplementary material for: A Keystone Ant Species Provides Robust Biological Control of the Coffee Berry Borer Under Varying Pest Densities
Source: PLoS One. 2015 Nov 12;10(11):e0142850. doi: 10.1371/journal.pone.0142850 (PMC4642973; doi:10.1371/journal.pone.0142850)

S1 Figure. Numerical Response. Shows mean branch ant activity (number of ants on or crossing onto branch/minute) (± SE) of *Azteca sericeasur* (on branches with ants only) 24 hours after CBB placement across the experimental CBB density treatments. The line shows a linear regression model of the data ($R^2 = 0.003$, $F = 0.005$, $p = 0.950$), which suggests there was no lasting numerical response of the ants to increased CBB density.

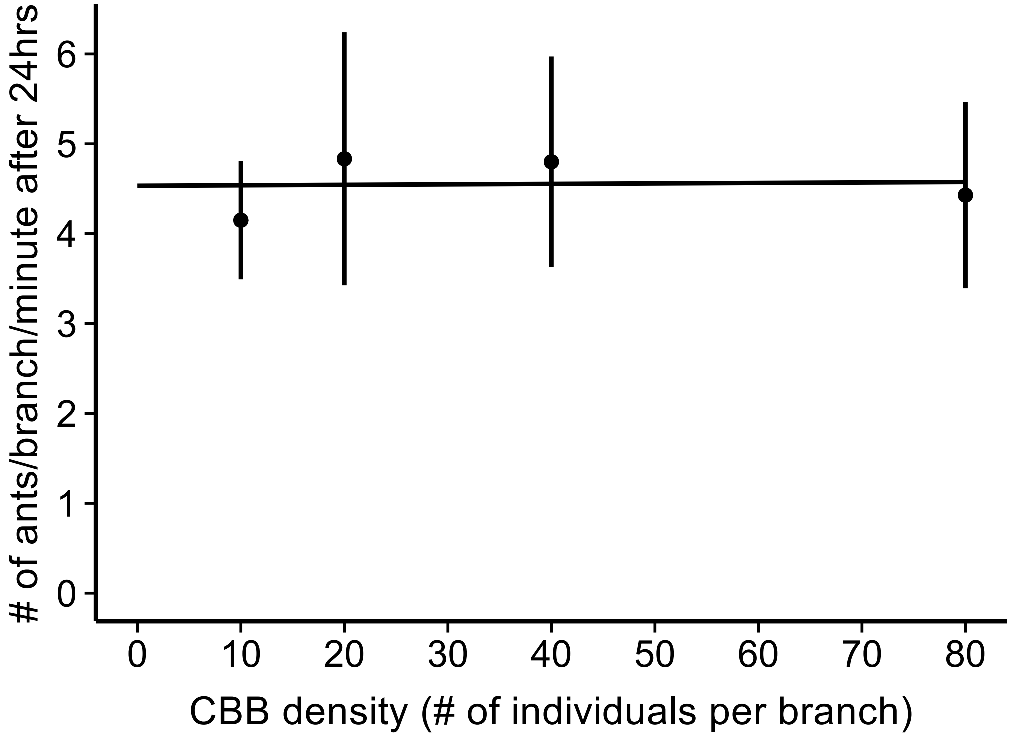

Supplement: S1 Fig — (PDF) [file pone.0142850.s002.pdf]
