# Supplementary material for: A Keystone Ant Species Provides Robust Biological Control of the Coffee Berry Borer Under Varying Pest Densities
Source: PLoS One. 2015 Nov 12;10(11):e0142850. doi: 10.1371/journal.pone.0142850 (PMC4642973; doi:10.1371/journal.pone.0142850)

S2 Figure. Branch Ant Activity. Shows mean branch ant activity (number of ants on or crossing onto branch/minute) (± SE) of *Azteca sericeasur* over the course of the experiment on branches with ants only. Data are separated by CBB density treatment (10, 20, 40, and 80 individuals), which is indicated above each bar plot. “Set up” is ant activity measured at the beginning of the experiment, before manipulating the plant. “CBB placement” is ant activity measured immediately before placing CBB on branches. “After 24 hrs” is ant activity measured 24 hours after CBB placement, at the end of the experiment. There was no statistical difference in mean branch ant activity by time (χ^2 = 3.290, p = 0.193), density treatment (χ^2 = 1.648, p = 0.649), or their interaction (χ^2 = 8.353, p = 0.213).

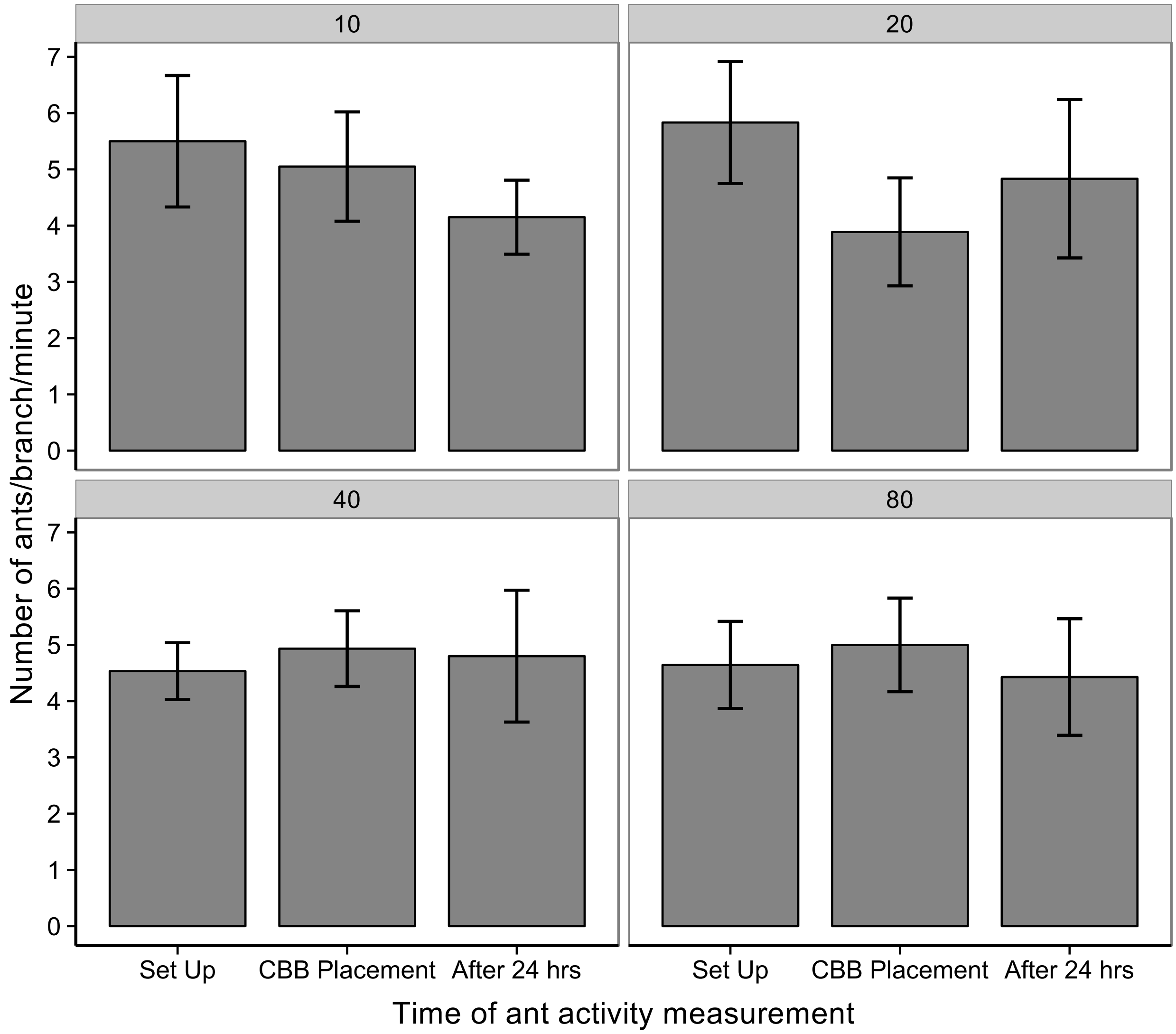

Supplement: S2 Fig — (PDF) [file pone.0142850.s003.pdf]
